# Case report: 17α− hydroxylase deficiency due to a hotspot variant and a novel compound heterozygous variant in the *CYP*17*A*1 gene of five Chinese patients

**DOI:** 10.3389/fped.2022.935191

**Published:** 2022-09-21

**Authors:** Jinying Li, Qiang Zhang, Jing Chen, Xingjiao Fu, Jingpin Yang, Lijun Liu

**Affiliations:** Department of Endocrinology, Genetics and Metabolism, Children’s Hospital of Hebei Province, Shijiazhuang, China

**Keywords:** 17α-hydroxylase deficiency, novel variant, twin, children, hypertension, hypokalemia

## Abstract

17α-Hydroxylase deficiency (17OHD) is a rare form of congenital adrenal hyperplasia caused by mutations in the *CYP17A1* gene. It is characterized by impaired adrenal and gonad steroid biosynthesis. Affected patients present with hypertension, hypokalemia, and disorders of sexual development. Here, we describe the genotypes and phenotypes of five patients from three families with this rare disease. Most patients had the hotspot variant, c.985_987delTACinsAA, in *CYP17A1*, which may be caused by a founder effect. However, the patients in our study were younger than the typical age of onset of 17OHD, and there was a pair of twins with the karyotypes 46, XX and 46, XY, but they both had a female phenotype. Meanwhile, we identified a novel compound heterozygous variant, c.1243+6T>G (p.Y329fs/splicing) in the *CYP17A1* gene.

## Introduction

Cytochrome P450 17α-hydroxylase (P450c17) is a polypeptide comprising of 508 amino acids. It is mainly distributed in the Leydig cells of the testes, follicular ovarian cells, and zona fasciculata and zona reticularis of the adrenal gland and is encoded by the CYP17A1 gene. P450c17 has dual functions: hydroxylation and lyase reactions (1), and is encoded by the *CYP17A1* gene. Mutations in the *CYP17A1* gene lead to a deficiency in 17α-hydroxylase/17,20-lyase (2). 17α-hydroxylase activity catalyzes the conversion of pregnenolone to 17α-hydroxypregnenolone and progesterone to 17α-hydroxyprogesterone. 17α-Hydroxypregnenolone and 17α-hydroxyprogesterone are then converted to dehydroepiandrosterone and androstenedione, respectively, by 17,20-lyase activity (3).

17α-Hydroxylase deficiency (17OHD) is an autosomal recessive disease, accounting for approximately 1% of congenital adrenal hyperplasia cases in newborns. The first case of 17OHD was described by Biglieri et al. (4) in a patient with a 46, XX karyotype who presented with hypertension and primary amenorrhea. Then, in 1970, the first 17OHD in a 46, XY individual with atypical genitalia, was reported by New (5). Since then, more than 500 cases of 17OHD have been reported in the literature (6).

The annual incidence of 17OHD is approximately 1 in 50,000 (7). 17OHD is caused by mutations in the cytochrome P450, family 17, subfamily A, polypeptide 1 (*CYP17A1*) gene, which encodes 17α-hydroxylase, a key enzyme for cortisol and sex hormone synthesis. Some *CYP17A1* mutations impair the enzyme activity and partially or completely affect adrenal and gonadal steroidogenesis. Patients with 17OHD show increased synthesis of 11-deoxycorticosterone (DOC) and corticosterone leading to mineralocorticoid excess (hypertension and hypokalemia) and decreased levels of sex steroids (dehydroepiandrosterone, androstenedione, testosterone, and estradiol) resulting in the disorder of sexual development in 46, XY and delayed puberty in both 46, XY and 46, XX (8).

17OHD is often associated with life-threatening complications such as hypokalemic period paralysis or hypertensive crisis. In addition, late diagnosis leads to medical and psychosocial implications due to delayed puberty. But, late diagnosis is not uncommon as this disease is often missed or misdiagnosed during childhood. In this report, we present the genetic and clinical characteristics of this rare disease in five patients from three families, including a pair of twins, and the discovery of a novel mutation. It is hoped to improve clinicians’ understanding of the disease.

## Patients and methods

### Ethics approval

The study was approved by the Institutional Ethics Committee of the Children’s Hospital of Hebei Province. The patients’ parents provided informed consent for participation in this study and the publication of the results.

### Patients

From January to September 2019, five patients from three kindreds were diagnosed with 17OHD after presenting to the Endocrinology and Genetic Metabolism Department of the Children’s Hospital of Hebei Province with different complaints. All patients were born to non-consanguineous parents and had no family history of chronic illnesses. Their ages ranged from 1 to 12 years, with a median age of 6 years.

In the first family, the index patient (patient 1) was a 12-year-old female-phenotype patient who presented with hypokalemic (1.8 mmol/l) weakness that was accompanied by hypertension (138/94 mmHg). She was born after an uneventful pregnancy and was the first child in the family. Her height was 142 cm and her weight was 44 kg. She had Tanner stage 1 breasts, pubic hair, and no axillary hair. Her external genitalia were female type, but with infantilism. She had three younger siblings, all with female external genitalia. All siblings of the index case were asymptomatic and no other family member had a similar history of hypertension and periodic paralysis. But, the third (karyotype 46, XY, patient 2) and fourth (karyotype 46, XX, patient 3) children were twins and had hypertension on examination (Table 1). At the age of 4 years, the proband had undergone surgery at the local hospital for an inguinal hernia on the left side. Regrettably, no pathological analysis of these tissues was performed.

**TABLE 1:** Clinical characteristics, biochemical, hormonal, and mutation findings of five 17α-hydroxylase deficiency cases.

Clinical information	Patient 1	Patient 2	Patient 3	Patient 4	Patient 5	Reference range
Age (years old)	12	6	6	6	1	
Social gender	Female	Female	Female	Female	Female	
External genital	Female	Female	Female	Female	Female	
Breast (Tanner grade)	1	1	1	1	1	
Height (cm) (SD)	142 (<–1 SD)	–	–	136 (2 SD)	–	
Blood pressure (mmHg)	138/94	136/98	124/83	169/103	Normal	
Karyotype	46, XY	46, XY	46, XX	46, XY	46, XY	
Pelvic ultrasound						
Uterus	Not find	Not find	33 × 3.2 × 2.6 mm	Not find	Not find	
Ovaries	Not find	Not find	Unclear	Not find	Not find	
Testis	Not find	Not find	Not find	Not find	In bilateral groin	
Pelvic MRI (uterus and ovaries)	Not find	–	–	–	–	
Adrenal MRI	–	–	–	Left adrenal nodular	–	
Adrenal CT	No abnormality	–	–	Left adrenal nodular	–	
Adrenal ultrasonography	No abnormality	No abnormality	No abnormality	No abnormality	No abnormality	
Clinical information	Patient 1	Patient 2	Patient 3	Patient 4	Patient 5	Reference range
Bone age (years)	8	–	–	6	–	
LH (IU/l)	16.0	0.742	<0.1	<0.1	4.92	<0.1
FSH (IU/l)	54.91	22.11	10.04	11.43	5.69	
E 2 (pg/ml)	<20.0	<18.5	<18.5	<18.5	<18.5	<18.5
T (ng/ml)	<0.20	<0.087	<0.087	<0.087	<0.087	<0.087
P (nmol/l)	3.17	19.33	16.35	28.65	2.64	
ACTH (pg/ml)	238	54.6	85.6	207	27.3	<46
Cortisol (8: 00) nmol/l	20.31	16.26	16.95	29.76	263.7	
17OHP (ng/ml)	0.2	–	–	0.34	0.57	<2.3
DHEA (ng/ml)	0.46	–	–	0.55	0.49	Male 0–8y 0.3–2.2 8–12y 0.5–5.5
DHT (pg/ml)	<0.17	–	–	35.23	19.86	40.5–355
AD (ng/ml)	<0.3	–	–	<0.3	<0.3	0.6–3.1
PRA (h)	0.86	–	–	0.89	–	supine position 0.15–2.33 upright position 1.31–3.95
ALD (pg/ml)	259.01	–	–	88.918	–	88.918
AMH (ng/ml)	>16.1	–	–	–	>14.70	
Clinical information	Patient 1	Patient 2	Patient 3	Patient 4	Patient 5	Reference range
INB (pg/ml)	67.8	–	–	–	363.39	
SRY	–	–	–	–	Positive	
Mutation	c.985_987 delTACinsAA	c.985_987 delTACinsAA	c.985_987 delTACinsAA	c.985_987 delTACinsAA c.1246C>T (p.R416C)	c.985_987 delTACinsAA c.1243+6T>G (splicing)	
Type	Homozygous	Homozygous	Homozygous	Compound heterozygous	Compound heterozygous	
Blood potassium (mmol/l)	1.87	3.43	3.55	3.24	4.02	3.5–5.5

ACTH, adrenocorticotropic hormone; F, cortisol; 17OHP, 17α-hydroxyprogesterone; DHEA, dehydroepiandrosterone; DHT, dihydrotestosterone; AD, androstenedione; PRA, Renin; ALD, aldosterone; AMH, anti-Müllerian hormone; INB, Inhibin B; LH, luteinizing hormone; FSH, follicle-stimulating hormone; E 2, estradiol; T, testosterone; P, progesterone; PRL, prolactin; SRY, sex-determining region Y.

In the second kindred, a 6-year-old child (patient 4) with female phenotype presented for incidentally diagnosed increased blood pressure during a school health check. Her weight and height were 49 kg (3 SDs above the mean) and 136 cm (2 SDs above the mean), respectively. The bone age of patient 4 was approximately 6 years. In kindred 3 (patient 5), the parents noted that the child had with a vulva, but no vagina. However, testicular masses were palpable in the respective inguinal regions. The child underwent surgical exploration, which revealed that a vagina was absent and right and left masses were confirmed to be testes.

### Hormonal measurements

Blood tests were performed according to standard methods. Blood hormone, Adrenocorticotropic hormone (ACTH), plasma renin activity (PRA), aldosterone (ALD), 17α-hydroxyprogesterone (17OHP) AMH (anti-Müllerian hormone), and inhibin B (INB) were measured by chemiluminescence; cortisol (F) was measured by electrochemiluminescence; dihydrotestosterone (DHT), and androstenedione (AD) were measured by liquid chromatography-mass spectrometry; dehydroepiandrosterone (DHEA), luteinizing hormone (LH), follicle-stimulating hormone (FSH), estradiol (E2), testosterone (T), progesterone (P), and prolactin (PRL) levels were measured by enzyme-linked immunosorbent assay (Table 1).

### Genetic analysis

With the consent of the patient’s parents, we collected the peripheral blood of the patients and their parents in three families. In addition, in family 1, peripheral blood of the proband’s sister was collected. Later, DNA was extracted, next-generation sequencing (NGS) sequencing was performed for the probands. The observed variants in the probands were confirmed and the family members were screened for the respective variants by Sanger’s method. A minimum of 3 μg DNA was used to prepare indexed Illumina libraries (MyGenostics, Beijing, China), according to the manufacturer’s protocol. The exons of genes associated with congenital adrenal hyperplasia (all the genes including ABCD1 AIRE ARMC5 CDKN1C CYP11A1 CYP11B1 CYP11B2 CYP17A1 CYP21A2 DHCR7 GK GK2 GLCCI1 GNAS H6PD HESX1 HSD11B1 HSD3B2 LHX4 MC2R MCM4 MEN1 MKS1 MRAP NNT NR0B1 NR3C1 NR5A1 PCSK1 PDE11A PDE8B POMC POR PRKACA PRKAR1A PROP1 REN RXRA RXRB SOX3 STAR TBX19 TP53 TXNRD2) were targeted *via* a gene capture strategy, using the GenCap custom enrichment kit (MyGenostics), according to the manufacturer’s protocol. The enriched libraries were sequenced on a NextSeq 500 sequencer (Illumina, San Diego, CA, USA) to generate paired-end reads of 150 bp. Single-nucleotide polymorphisms, insertions, and deletions were identified using Genome Analysis Toolkit software.

## Results

### Clinical characteristics

All five patients had a female external genitalia phenotype, and four of them had hypertension and hypokalemia. At the time of admission, the index patient (1a, patient 1) of the first family was diagnosed with hypokalemia (1.8 mmol/l) and elevated blood pressure (138/94 mmHg). The second child of family 1, had no abnormal manifestations. The third (1b, patient 2) and fourth (1c, patient 3) children of the family were 6-year-old twins. Both patients 2 and 3 had elevated blood pressure but were asymptomatic. Their parents, grandparents or maternal grandparents had normal blood pressure and with no history of abnormal sexual development.

In kindred 2 (patient 4), a 6-year-old child was found to have hypertension during a physical exam and a weight and height of 49 kg (3 SDs above the mean) and 136 cm (2 SDs above the mean), respectively. The bone age of patient 4 was approximately 6 years. In kindred 3 (patient 5), after the child was born, the parents noted that the child’s external genitalia presented with a vulva, but no vagina. However, testicular masses were palpable in inguinal region. The child underwent surgical exploration, which revealed that a vagina was absent and mention right and left masses were testis.

### Genetic analysis

**FIGURE 1:**
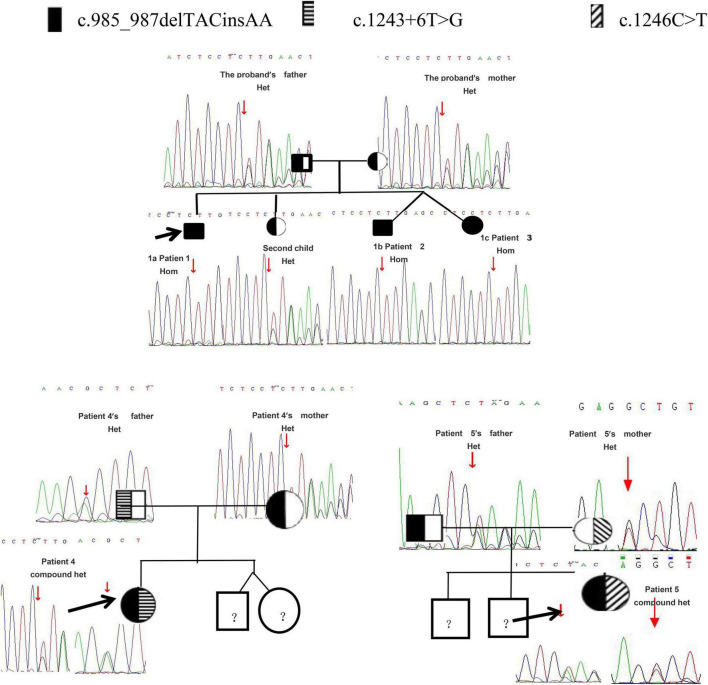
The pedigree and gene sequencing results of five patients.

Sequencing of CYP genes in the proband (patient 1), who had a karyotype of 46, XY, identified a homozygous mutation, c.985_987delTACinsAA (p.Y329fs), within exon 6 of the *CYP17A1* gene. The mutation was reported in The Human Gene Mutation Database. The father, mother, and the second child of this family were all found to be heterozygous carriers of the same mutation, and the twins had the same homozygous mutation as the proband (Figure 1). In kindred 2, sequencing results revealed a compound heterozygous mutation in the *CYP17A1* gene: c.1246C>T (p.R416C), c. 985_987delTACinsAA (p. Tyr329fs). In kindred 3, the following compound heterozygous mutation of the *CYP17A1* gene was identified: c.1243+6T>G (splicing), c. 985_987delTACinsAA (p. Tyr329fs),several analysis tools, including SIFT, PolyPhen_2, Mutation Taster, GERP++, and REVEL revealed that it was a variant of unknown significance, but PM3 as a cause of recessive hereditary disease, when existing with another disease-causing mutation in trans (i.e., forming a complex heterozygous mutation with another disease-causing mutation). There was no correlation report for this locus in the HGMD database and no pathogenicity notation in the ClinVar database. Parental analysis showed that the patient’s father carried the c.985_987delTACinsAA (p.Y329fs) mutation and the mother carried the c.1243+6T>G (splicing) mutation.

## Discussion

17OHD is characterized by impaired adrenal and gonad steroid biosynthesis, a complete deficiency of enzyme activity resulting in decreased adrenal and gonadal sex steroid synthesis, a decrease in cortisol synthesis, augmented adrenocorticotropic hormone (ACTH) secretion, and overstimulation of the 17-deoxy pathway leading to increased progesterone, corticosterone, and DOC synthesis (9). Patients present typically with hypertension and hypokalemia. Females can also present with hypergonadotrophic hypogonadism, delayed puberty, and primary amenorrhea. Males may have ambiguity or female external genitalia (10).

In this study, four of the 17OHD patients had karyotypes of 46, XY, and one had a karyotype of 46, XX, but they all presented with female external genitalia. Fetal testes normally express P450c17 early in the gestational period to produce testosterone, which promotes the development of external male genitalia. However, in 17OHD, in early fetal life, the lack of testosterone contributes to the development of female external genitalia in males. The lab results for these patients showed low levels of testosterone, dehydroepiandrosterone, and androstenedione. Consistent with these results, they presented with hypergonadotropic hypogonadism characterized by high levels of serum luteinizing hormone (three out of five), and follicle-stimulating hormone. Progesterone was elevated in all patients (that was not coupled with marked elevation of 17OHP) favoring a hormonal diagnosis of 17OHD. Hypokalemia inhibits the secretion of PRA by the zona globules of the adrenal gland, and therefore their PRA were reduced.

The anti-Müllerian hormone (AMH) and inhibin B levels of patients 1 and 5 suggested that they had normal Sertoli cell function. Normal production of AMH in the testes of 17OHD patients leads to the regression of Müllerian structures. Previous studies have reported that 46, XY individuals with 17OHD have absent internal Müllerian structures (uterus, fallopian tubes, and upper third of the vagina) and pseudohermaphroditism. This was also true for the four 46, XY individuals in this study. Human chorionic gonadotrophin stimulation test results were abnormal in patients 1 and 5, with no increase in testosterone levels consistent with defective androgen biosynthesis in 17OHD (Table 2).

**TABLE 2:** Human chorionic gonadotrophin stimulation result of the patients.

Patient	T1/T2 (nmol/l)	T2 (nmol/l) /DHT (pg/ml)
Patient 1	<0.20/<0.20	<0.20 nmol/l/<0.17 pg/ml
Patient 5	<0.087/<0.087 nmol/l	<0.087 nmol/l/25.53 pg/ml

Hypertension and hypokalemia are the major features of 17OHD. Four out of five of the patients in this study presented with varying degrees of hypertension and hypokalemia, and increases in blood pressure with age. Previous studies have suggested that the absence of 17-hydroxylase enzyme activity drives the overproduction of 11-DOC and corticosterone, leading to mineralocorticoid effects (10) and consequently, fluid and sodium retention, hypertension, and hypokalemia. Research suggests that the newborn kidney is rather insensitive to mineralocorticoids, and thus, manifestations of excess levels of the mineralocorticoid DOC tend not to occur in infancy (8). However, gradually, hypertension and hypokalemia caused by DOC excess may show a typical manifestation. Another possibility is that even young children with 17OHD may have high blood pressure, but it is not often measured (11). Such patients are usually diagnosed during adolescence, owing to delayed puberty, hypertension, and hypokalemia (12). Therefore, children may easily escape diagnosis and only be diagnosed at puberty. In our study, the blood pressure of the 12-year-old child was higher than that of the 6-year-old child, while the blood pressure of the 1-year-old child was not elevated.

In kindred 1, we found that the twin with the 46, XY karyotype (patient 2) had higher blood pressure and lower blood potassium levels than the twin with the 46, XX karyotype (patient 3), despite having the same homozygous mutations in CYP17A1. Therefore, we conclude that the response to increased DOC levels may be weak in females (13, 14).

The first child in kindred 1 presented with fatigue because of hypokalemia, but none of the other patients had symptoms of hypokalemia, even though their serum potassium levels were low or lower than the limit of normal. Not all the patients had hypertension and hypokalemia, as patient 5 had normal blood pressure and normal serum potassium levels. Research suggests that 10–15% of 17OHD patients do not have hypertension or hypokalemia (14) and the extent of hypertension and hypokalemia varies from case to case. Thus, 17OHD should not be ruled out because of the absence of hypertension or remarkable hypokalemia (15).

**FIGURE 2:**
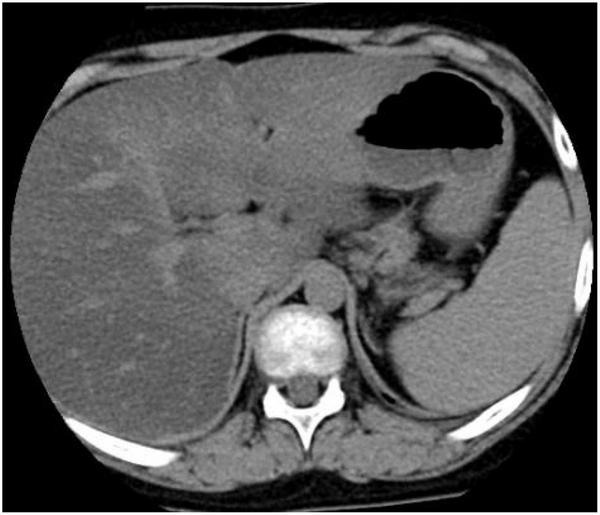
Computed tomography (CT) scan of the adrenal gland showed nodular thickening of the left adrenal junction of patient 4.

In 17OHD patients, the synthesis of cortisol is decreased, and in response, the pituitary gland increases the production of ACTH, which may lead to bilateral adrenocortical hyperplasia (6). Furthermore, magnetic resonance imaging (MRI) or computed tomography (CT) scans of the adrenal glands may demonstrate adrenal hyperplasia. In this study, adrenal MRI and CT scans of patient 4 showed nodules at the junction of the adrenal gland, (Figure 2) but adrenal ultrasonography showed no abnormalities, indicating that adrenal MRI is superior to adrenal ultrasonography in the diagnosis of adrenal hyperplasia.

17-OHD is an autosomal recessive disease involving the *CYP17A1* gene located on chromosome 10q24.3 (16). More than 100 mutations in the *CYP17A1* gene have been found to be associated with combined 17-hydroxylase/17,20-lyase deficiency (OMIM 202110) (2). In the present report, genetic analysis identified three *CYP17A1* gene mutations, including one homozygous frameshift mutation (Y329fs) in three patients from kindred 1 (patients 1, 2, and 3), a compound heterozygous mutation (p.Y329fs and p.R416C) in patient 4 from kindred 2, and a splice-site mutation (c.1243+6T>G) in patient 5 from kindred 3.

In kindred 1, genetic analysis revealed that all the patients were homozygous for the mutation, c.985_987delTACinsAA (p.Y329fs) in exon 6 of the *CYP17A1* gene. This mutation is one of the most commonly mutated sites in Chinese 17OHD patients. Wang et al. (6) reported that 70 of 181 (38.6%) Chinese 17OHD patients that underwent genetic testing had the c.985_987delTACinsAA185 (p.Y329K) mutation, and another study reported that this mutation is present in 60.8% of 17OHD patients (17). Thus, this locus appears to be a mutational hot spot (8) and is thought to be the result of founder effects (10). The homozygous mutation c.985_987delTACinsAA (p.Y329fs), is a frameshift mutation that introduces a premature stop codon at nucleotide 329 in exon 6, as well as a 1-base deletion and a 1-base transversion (TAC → AA) at codon 329, causing the premature truncation of protein synthesis at codon 417 (1). Therefore, the pivotal functional domain of the heme-binding region is lost, resulting in the complete loss of 17-hydroxylase/17,20-lyase activity (2) and blockage of the synthesis of 17α-hydroxypregnenolone and 17α-hydroxyprogesterone by 17α-hydroxylase activity. This results in the excessive synthesis of the mineralocorticoid precursors which leads to hypertension, hypokalemia, and markedly decreased sex hormone (testosterone and estradiol) levels, which causes sexual infantilism (18).

Patient 4 of kindred 2 had the frameshift mutation c.985_987delTACinsAA at codon 329 in exon 6 and the missense mutation 1246C>T at exon 8 in the *CYP17A1* gene, which substitutes arginine at 416 with cysteine. These compound heterozygous mutations resulted in very weak activities of 17α-hydroxylase and 17,20-lyase (19).

Patient 5 of kindred 3 carried compound heterozygous mutations consisting of the frameshift mutation c.985_987delTACinsAA (p.Y329fs) and the novel splice-site mutation c.1243+6T>G, in exon 7 of *CYP17A1*. This mutation was presumed to cause changes in the amino acid sequence and it appeared to cause 17OHD.

The clinical features of the patients in this study and their biochemical assay results showing low levels of estradiol, testosterone, and androstenedione combined with high levels of gonadotropins and progesterone confirmed the clinical diagnoses of 17OHD (Tables 1, 3). We evaluated genetic variants that impaired the enzymatic function of 17α-hydroxylase, and novel compound heterozygous mutations were identified as pathogenic. These novel mutations expand the spectrum of pathogenic *CYP17A1* gene mutations.

**TABLE 3:** Gonadotropin-releasing hormone stimulation result of the patient 5.

Patient	LH1/FSH1 (IU/l)	LH2/FSH2 (IU/l)	LH3/FSH3 (IU/l)
Patient 5	58.55/14.27	82.52/21.39	100/27.67

Two of our patients with elevated blood pressure (patients 1 and 4) were treated with hydrocortisone at doses of 15–25 mg/m^2^/d), after which their blood pressure returned to normal, and their serum potassium level returned to within the normal range after approximately 20 days. Currently, after approximately 3 years of follow up, these patients are still taking a physiological dose of hydrocortisone, their blood pressure is stable, and their serum potassium levels are normal. However, the other patients were not treated because their symptoms were not obvious and the parents refused treatment.

The study had a few limitations. Firstly, some medical records were not comprehensive enough, which is not conducive to further understanding of the disease. Secondly, pedigree analysis was incomplete in some patients.

In conclusion, 17OHD is a rare disease. The majority of 17OHD patients remain asymptomatic until adolescence, making early diagnosis relatively difficult. The present report suggests that 17OHD should be considered in the differential diagnosis of children with hypertension and/or hypokalemia, atypical genitalia, or adolescents with delayed puberty. We also suggest that blood pressure measurement should be part of regular school health checks which may help in the early diagnosis of such rare disorders.

## Data availability statement

The datasets for this article are not publicly available due to concerns regarding participant/patient anonymity. Requests to access the datasets should be directed to the corresponding author.

## Ethics statement

The studies involving human participants were reviewed and approved by the Children’s Hospital of Hebei province. Written informed consent to participate in this study was provided by the participants’ legal guardian/next of kin. Written informed consent was obtained from the individual(s), and minor(s)’ legal guardian/next of kin, for the publication of any potentially identifiable images or data included in this article.

## Author contributions

JL wrote the manuscript under the direction of LL. JC, JY, QZ, and XF analyzed the experimental results together. All authors contributed to the article and approved the submitted version.
